# Regulation of fucose and 1,2-propanediol utilization by *Salmonella enterica* serovar Typhimurium

**DOI:** 10.3389/fmicb.2015.01116

**Published:** 2015-10-12

**Authors:** Lena Staib, Thilo M. Fuchs

**Affiliations:** Lehrstuhl für Mikrobielle Ökologie, Zentralinstitut für Ernährungs- und Lebensmittelforschung Institute for Food and Health, Technische Universität MünchenFreising, Germany

**Keywords:** propanediol, *Salmonella enterica* serovar Typhimurium, metabolism, fucose, anaerobic growth

## Abstract

After ingestion, *Salmonella enterica* serovar Typhimurium (*S*. Typhimurium) encounters a densely populated, competitive environment in the gastrointestinal tract. To escape nutrient limitation caused by the intestinal microbiota, this pathogen has acquired specific metabolic traits to use compounds that are not metabolized by the commensal bacteria. For example, the utilization of 1,2-propanediol (1,2-PD), a product of the fermentation of L-fucose, which is present in foods of herbal origin and is also a terminal sugar of gut mucins. Under anaerobic conditions and in the presence of tetrathionate, 1,2-PD can serve as an energy source for *S.* Typhimurium. Comprehensive database analysis revealed that the 1,2-PD and fucose utilization operons are present in all *S. enterica* serovars sequenced thus far. The operon, consisting of 21 genes, is expressed as a single polycistronic mRNA. As demonstrated here, 1,2-PD was formed and further used when *S.* Typhimurium strain 14028 was grown with L-fucose, and the gene *fucA* encoding L-fuculose-1-phosphate aldolase was required for this growth. Using promoter fusions, we monitored the expression of the propanediol utilization operon that was induced at very low concentrations of 1,2-PD and was inhibited by the presence of D-glucose.

## Introduction

Members of the genus *Salmonella* are globally distributed and comprise 2,500 serovars described thus far. They infect cold- and warm-blooded animals as well as humans, and their transmission generally follows the fecal–oral route by consumption of contaminated foods, mostly of animal source. In Germany, salmonellosis (non-typhoidal *Salmonella* gastroenteritis) is the second most common form of bacterial gastroenteritis, with 18,986 reported cases in 2013 and an estimated 80–90% unreported cases (Koch-Institut, 2014). In Europe, 91,034 salmonellosis cases were reported in 2012 (European Centre for Disease Prevention and Control, 2014). In the USA, *Salmonella* causes an estimated one million illnesses annually (Centres for Disease Control and Prevention, 2013). A major concern for public health is the emergence of *Salmonella* strains resistant to antimicrobials (Majowicz et al., 2010).

*Salmonella enterica* serovar Typhimurium (*S.* Typhimurium) causes non-typhoidal gastroenteritis in humans and typhoid-like disease in mice (Tsolis et al., 1999; Zhang et al., 2003). Expression of genes located on the so-called *Salmonella* pathogenicity islands (SPI) 1 and 2 are important during infection by enabling the invasion of epithelial cells and the intracellular replication and survival of *S.* Typhimurium (Galan and Ginocchio, 1994; Ochman et al., 1996). The acquisition of nutrients within the infected host is crucial for enteric pathogens, because they need energy as well as carbon and nitrogen sources in order to grow within host compartments, to compete against commensals, to colonize the epithelium, to produce virulence factors, to withstand the hosts’s immune responses, and to transmit themselves to other hosts or the environment (Staib and Fuchs, 2014). As revealed previously, more than 60 compounds can serve as substrates for *S.* Typhimurium (Gutnick et al., 1969). However, it remains obscure which substrates are available or used during multiplication in the intestinal lumen and subsequent infection. Many enteric pathogens are equipped with specific metabolic pathways that play predominantly a role during infection to overcome nutrient limitations imposed by the host (Fuchs et al., 2012; Abu Kwaik and Bumann, 2013). Examples in *S.* Typhimurium are the gene clusters responsible for sialic acid, *myo*-inositol, and ethanolamine utilization (Staib and Fuchs, 2014). Proliferation of *S.* Typhimurium was impaired in gnotobiotic mice by a lack of sialic acids due to co-colonization with a sialidase-deficient *Bacteroides thetaiotaomicron* strain (Ng et al., 2013). The *iol* genes responsible for the degradation of *myo*-inositol (Kröger and Fuchs, 2009; Kröger et al., 2011; Rothhardt et al., 2014) are thought to contribute to the virulence of *S.* Typhimurium in mice, pigs, chickens, and calves (Lawley et al., 2006; Carnell et al., 2007; Chaudhuri et al., 2009, 2013). Growth attenuation has been reported in food, nematodes, and mice for *S.* Typhimurium deficient in ethanolamine utilization (Stojiljkovic et al., 1995; Srikumar and Fuchs, 2011; Thiennimitr et al., 2011). Recently, it was shown that tetrathionate is formed in the inflamed intestine from the reaction of reactive oxygen species (ROS) and sulfur compounds such as thiosulfate, a finding that linked anaerobic respiration and ethanolamine degradation with *S.* Typhimurium proliferation in the gut (Winter et al., 2010). Analysis of *in vivo*-induced genes and competitive index studies identified the capability of *S*. Typhimurium to degrade 1,2-propanediol (1,2-PD) relevant for infection (Conner et al., 1998). This result was confirmed by the findings that propanediol utilization (*pdu*) genes responsible for 1,2-PD are induced in murine and human cells, and that a lack of *pdu* genes results in reduced replication in macrophages (Heithoff et al., 1999; Klumpp and Fuchs, 2007).

*Salmonella enterica* serovar Typhimurium is also capable of producing cobalamin (vitamin B_12_) under anaerobic conditions (Roth et al., 1996). The *cob/cbi* operon, responsible for *de novo* synthesis of cobalamin, and the *pdu* operon comprising 23 genes encoding enzymes and a polyhedral body, are located side by side on the genome of *S.* Typhimurium (Jeter, 1990; Bobik et al., 1999). The expression of both operons is positively regulated by PocR and by two global regulators, namely the cAMP receptor protein (CRP) and ArcA/ArcB for anoxic respiratory control (Bobik et al., 1992; Rondon and Escalante-Semerena, 1996; Ailion and Roth, 1997). Indeed, cobalamin plays a role in 1,2-PD degradation by *S.* Typhimurium as it is a cofactor of propanediol dehydratase, the first enzyme of this catabolic pathway (Chen et al., 1994; Bobik et al., 1997; Walter et al., 1997). 1,2-PD can serve as a carbon and energy source for *Clostridium glycolicum* and *Klebsiella pneumoniae* in a cobalamin-dependent manner under anaerobic conditions (Gaston and Stadtman, 1963; Toraya et al., 1979). *S.* Typhimurium was able to grow with 1,2-PD in the presence of this vitamin under aerobic conditions (Jeter, 1990). Growth of *S.* Typhimurium with 1,2-PD in the absence of oxygen, however, was observed only when tetrathionate, which serves as a terminal electron acceptor for anaerobic respiration of 1,2-PD, was added to the medium (Price-Carter et al., 2001). 1,2-PD is the fermentation end-product of bacterial growth with L-fucose and/or L-rhamnose (Daniel et al., 1998). While *Escherichia coli* is unable to further metabolize 1,2-PD when kept under anaerobic conditions, this compound vanishes from the *S.* Typhimurium culture medium suggesting its further utilization by this pathogen (Obradors et al., 1988).

The two sugars L-fucose and L-rhamnose are frequently found in the carbohydrate moieties of mucosal glycoconjugates, herbal cell walls, and bacterial exopolysaccharides (Sampson and Bobik, 2008). L-fucose, comprising 4–14% of the oligosaccharide content of mucins, is found mainly as a terminal sugar of the oligosaccharide chains linked to the mucin protein backbone (Muraoka and Zhang, 2011). Thus, mucus is the main source of gastrointestinal L-fucose provided by the enzymatic activities of *Bacteroides* (Keeney and Finlay, 2013). Degradation of L-fucose monomers is not restricted to commensal bacteria such as *Bacteroides*. The genetic determinants for L-fucose utilization are also found in the genomes of many enteropathogens (Staib and Fuchs, 2014), although to date, experimental confirmation of fucose utilization is largely lacking. Fermentation of L-fucose and subsequent 1,2-PD secretion have been described for *E. coli* (Cocks et al., 1974), *K. pneumoniae*, and *S.* Typhimurium (Badia et al., 1985). More recently, proteomic and glycomic evidence has been provided that *S*. Typhimurium takes advantage of fucose metabolization while growing in the mouse gut (Deatherage Kaiser et al., 2013).

In this study, we investigated genetic determinants of *S.* Typhimurium with a role in the utilization of mucus-derived sugar components. We determined the distribution of *pdu* and *fuc* genes among salmonellae, and monitored promoter activities of both operons during all growth phases using luciferase reporter fusions. The results suggest a role of fucose and 1,2-PD utilization by *S.* Typhimurium in the environment and during infection.

## Materials and Methods

### Bacterial Strains, Plasmids, and Growth Conditions

Bacterial strains and plasmids used in this study are listed in **Table 1**. *S.* Typhimurium and *E. coli* strains were grown at 37°C in lysogeny broth (LB) medium (10 g/l tryptone, 5 g/l yeast extract, and 5 g/l NaCl) or on LB-agar (LB-medium supplemented with 1.5% [w/v] agar). Liquid cultures were inoculated using a single colony or a 1:1,000 dilution of an overnight culture, and shaken at 180 rpm. If appropriate, the following antibiotics were used: ampicillin (100 μg/ml), chloramphenicol (20 μg/ml), kanamycin (50 μg/ml), nalidixic acid (20 μg/ml), or tetracycline (12 μg/ml).

**Table 1:** Strains and plasmids used in the study.

Strain	Relevant genotype or characteristics	Reference
***Salmonella enterica* serovar Typhimurium (*S.* Typhimurium)**
14028 (Nal^R^)	Spontaneous mutation of strain ATCC 14028 on nalidixic acid	This study
14028 P_*pduA*_::*lux*	Genomic integration of pUTs *lux*-Cm^R^ in Nal^R^ background	This study
14028 P_*fucO*_::*lux*	Genomic integration of pUTs-*lux*-Cm^R^ in Nal^R^ background	This study
14028 Δ*pduC*	Deletion of *pduC* in Nal^R^ background	This study
14028 Δ*fucA*::*kanR*	Kan^R^ insertion mutant in place of *fucA*	This study
***Escherichia coli***
S17.1 λpir	λ-pir lysogen of S17.1 (Tp^R^, Strept^R^, Spec^R^ *thi pro hsdR^-^M^+^ recA* RP4::2-Tc::Mu Km::Tn17)	Simon et al., 1983
**Plasmids**
pUTs-*lux* (Cm^R^)	Suicide vector, promoterless *luxCDABE* genes, R6K *ori*, *oriT* (RP4); Cm^R^	Starke et al., 2013
pUTs-*gfp* (Cm^R^)	As above, promoterless *gfp*, R6K *ori*, *oriT* (RP4); Cm^R^	Starke et al., 2013
pKD46	λ Red recombinase expression plasmid, *oriR*101/*repA*101(ts), and P_*araB*_-*gam*-*bet*-*exo*, Amp^R^	Datsenko and Wanner, 2000
pKD4	*pir*-dependent, FRT-recognition sites; Kan^R^	Datsenko and Wanner, 2000
pCP20	FLP-recombinase plasmid; Cm^R^, Amp^R^	Datsenko and Wanner, 2000
pBR322	Cloning vector; Amp^R^, Tet^R^	Bolivar et al., 1977
pGreenTIR	GFP-cloning vector, translation initiation region (TIR), P_*lac*_-TIR-*gfp*; Amp^R^	Miller and Lindow, 1997
pUTs-P_*pduA*_::*lux* (Cm^R^)	Cloning of promoter region P*_*pduA*_* in front of *luxCDABE* via *Sac*I and *Kpn*I; Cm^R^	This study
pUTs-P*_*fucO*_*::*lux* (Cm^R^)	Cloning of promoter region P*_*fucO*_* in front of *luxCDABE* via *Sac*I and *Sma*I; Cm^R^	This study
pUTs-P*_*pduA*_*::*gfp* (Cm^R^)	Cloning of promoter region P*_*pduA*_* in front of *gfp* via *Not*I; Cm^R^	This study
pBR-*pduC*	Complementing plasmid, *Pvu*I and *Ase*I used for cloning of *pduC* into pBR322; Tet^R^	This study
pBR-*fucA*	Complementing plasmid, *Pst*I and *Ahd*I used for cloning of *fucA*; Tet^R^	This study

To test growth on single carbon sources, *S.* Typhimurium strain 14028 was cultivated in Vogel–Bonner no-carbon E medium (VB-NCE; 0.82 mM MgSO_4_, 0.0574 mM K_2_HPO_4_, and 16.74 mM NaNH_4_HPO_4_) supplemented with trace elements (0.3 mM CaCl_2_, 0.1 mM ZnSO_4_, 0.045 mM FeSO_4_, 0.2 mM Na_2_Se_2_O_3_, 0.2 mM Na_2_MoO_4_, 2 mM MnSO_4_, 0.1 mM CuSO_4_, 3 mM CoCl_2_, and 0.1 mM NiSO_4_; Vogel and Bonner, 1956; Price-Carter et al., 2001). To enhance initial growth, 0.2% (w/v) yeast extract (VB-NCE-YE) was added. 40 mM Na-tetrathionate was supplemented for anaerobic or 200 nM cyano-cobalamin for aerobic cultivation (Price-Carter et al., 2001). Unless otherwise stated, carbon sources were used in the following concentrations: 25 mM 1,2-PD, 25 mM L-fucose (fucose), and 27.8 mM D-glucose (glucose). Liquid media were inoculated using a final dilution of LB-overnight cultures of 1:250. For anaerobic cultivation (without shaking), media without tetrathionate were stored in an anaerobic chamber (A35, DonWhitley, Shipley, UK) in an atmosphere of 80% N_2_, 10% CO_2_, and 10% H_2_ overnight, before tetrathionate was added. To monitor the optical density at wavelength 600 nm (OD_600_) of a bacterial culture and, if applicable, its bioluminescence in relative light units of luminescence at OD_490_ (RLU_490_), samples were transferred in triplicate into 96-well microtiter plates with 200 μl of medium per well, and measured with the multilable plate reader Wallac Victor^3^ (Perkin Elmer, Waltham, MA, USA) as described previously (Kröger and Fuchs, 2009).

### General Molecular Techniques

Standard protocols (Sambrook and Russell, 2001) or manufacturer’s instructions were applied for manipulation and isolation of DNA and plasmids. For polymerase chain reaction (PCR) isolated DNA, plasmid DNA, cDNA, or single colonies dissolved in H_2_O served as templates. *Taq* polymerase (Schauer, 2010) was used with the following program: 95°C for 5 min and 35 cycles at 95°C for 10 s; annealing at temperatures chosen according to the primers for 30 s; elongation periods at 72°C chosen according to amplicon size; and a final elongation step at 72°C for 8 min. Primers for PCR were purchased from MWG-Biotech (Ebersberg, Germany). Amplification mixtures were loaded on agarose gels using GeneRuler^TM^ DNA ladder mix (Thermo Scientific, Braunschweig, Germany) as a size reference. *E. coli* strain S17.1 cells were used as the donor for conjugational transfer of pUTs vectors to *S.* Typhimurium (Simon et al., 1983).

### Microscopy

Bacteria were investigated for green fluorescent protein (GFP) expression using an Olympus BX-51 fluorescence microscope (Olympus, Hamburg, Germany). Analysis was performed using the F-View Soft Imaging System and the software cell^F^ at 1,000× magnification. Bacteria were concentrated after sampling by centrifugation and kept on ice until observation of aliquots using bright-field microscopy and the GFP fluorescence channel. To obtain three-dimensional pictures, an inverted confocal microscope (IX81, Olympus) was used at 400× magnification. Z-stacks were recorded and reconstructed with Volocity 6.0 software (Perkin Elmer).

### Construction of Mutants and Plasmids

To generate kanamycin-insertion mutants and non-polar gene deletions, we applied the method established by Datsenko and Wanner (2000). Briefly, vector pKD46 encoding the λ Red recombinase system was transferred into strain 14028 by electroporation. Cells were then transformed with a PCR fragment harboring the kanamycin cassette and FRT sites amplified from pKD4 as well as extensions of 50 bp representing the 3′- and 5′-ends of the target gene. Residues of 18 bp on the 5′- and 36 bp on the 3′-end of the gene were left to avoid damage to possible promoter structures (Link et al., 1997). Homologous recombination was confirmed by kanamycin selection. To circumvent possible illegitimate recombination effects, the kanamycin insertion was transferred to 14028 by phage transduction (Maloy, 1990) as follows: an overnight culture of 14028 harboring the kanamycin-resistance cassette was diluted 1:100 into LB-medium. At OD_600_ = 0.2, 5 ml of the culture was transferred into a glass tube, and 5 μl of a P22 suspension were added. The mixture was incubated for 6 h at 37°C without shaking. Then, 50 μl chloroform was added, and the suspension was stored at 4°C for 2 h. The cell debris was pelleted at 9,000 × *g* and 4°C for 10 min. The supernatant was filter sterilized with a 0.2-μm pore filter, and 200 μl of an overnight culture of strain 14028 was mixed with 10 μl of the phage lysate. After 1 h incubation at 37°C without shaking, the suspension was plated on selective agar-plates. Bacteria were cultivated on green indicator plates to isolate phage-free colonies. Vector pCP20 was used to eliminate the Kan^R^-cassette (Datsenko and Wanner, 2000). Deletions were confirmed by PCR and DNA sequencing (GATC, Konstanz, Germany).

To complement gene deletions, the coding sequences of the genes were cloned into pBR322. Gene *pduC* without a promoter was ligated into the *Pvu*I/*Ase*I restricted plasmid, thus exploiting the ß-lactamase (*bla*) promoter. For Δ*fucA* complementation, the promoter region of 500 bp upstream of *fucA* was amplified together along with the gene, and cloned into pBR322 via *Pst*I/*Ahd*I. Plasmids were verified by sequencing.

### Generation of Reporter Strains

To generate chromosomal fusions of promoter regions to the promoterless reporter genes *luxCDABE* or *gfp*, fragments located 500 bp upstream of the target genes *pduA* and *fucO* were amplified by PCR and cloned into the plasmids pUTs-*lux* and pUTs-*gfp* (see Supplementary Table S1 for primers). The ligated plasmids were transformed into *E. coli* S17.1, and putative positive clones were selected on LB-agar with chloramphenicol (20 μg/ml) and identified by PCR. Conjugation was then performed to transfer the recombinant plasmids to *S.* Typhimurium. Transformants were selected on LB-agar supplemented with nalidixic acid and chloramphenicol, or on chloramphenicol-containing Brilliance *Salmonella* agar (Oxoid, Wesel, Germany). Successful integration of the plasmid into the genome of *S.* Typhimurium strains was confirmed by PCR.

### Isolation of DNA and RNA, and cDNA Synthesis

To isolate bacterial genomic DNA, 1.5 ml of an overnight culture were centrifuged. After resuspension of the bacterial pellet in 400 μl lysis solution (100 mM Tris-HCl pH 8.0, 5 mM ETDA, and 200 mM NaCl), 100 μl lysozyme (10 mg/ml) was added. The suspension was stored on ice for 15 min and incubated over night at 55°C after addition of 10 μl 10% (w/v) sodium dodecyl sulfate (SDS) and 5 μl proteinase K (10 mg/ml). Then, 500 μl 2-propanol was added, and the DNA was sedimented by centrifugation. The supernatant was discarded, and the DNA was washed with 70% (v/v) ethanol. After a further centrifugation step, the DNA pellet was air-dried and dissolved in 150 μl H_2_O and 1 μl RNase (10 mg/ml).

For RNA isolation (Chomczynski and Sacchi, 1987), strain 14028 was grown in 50 ml VB-NCE-YE supplemented with either glucose or 1,2-PD to an OD_600_ of 2.0. The bacteria were harvested in 50 ml reaction tubes and centrifuged at 3,220 × *g* at 4°C for 10 min. The supernatant was discarded, and the pellet was dissolved in 1 ml TRIZOL (Invitrogen, Karlsruhe, Germany) by gentle mixing. The TRIZOL/RNA solution was extracted with 400 μl of chloroform, and the aqueous phase was mixed with 450 μl of 2-propanol. After 30 min at room temperature (RT) and a further centrifugation step, the supernatant was removed, and the pellet was washed with ethanol and resuspended in 25 μl RNase-free water (1 ml diethylpyrocarbonate in 1 l H_2_O). Forty micrograms of nucleic acids were then dissolved in 79 μl of RNase-free water and incubated at 65°C for 5 min. The RNA was kept on ice for 5 min, and 10 μl of 10× DNase buffer and 10 μl of DNase (1 U/μl) were added. After incubation at 37°C for 75 min, the sample was mixed with 100 μl Roti-Aqua-P/C/I and centrifuged at 15°C for 12 min at 17,000 × *g*. The aqueous phase was transferred to a 1.5-ml reaction tube. A 2.5-fold volume of a 1:30 mixture of ethanol and 3 M Na-acetate (pH 6.5) was added to the RNA sample. The tube was incubated overnight at -20°C and then centrifuged at 17,000 × *g* and 4°C for 30 min. The pellet was washed with 70% (v/v) ethanol and dissolved in 40 μl RNase-free water. The RNA concentration and quality was determined with a NanoDrop-1000 (Thermo Scientific). The qSkript^TM^ cDNA SuperMix of Quanta biosciences (Gaithersburg, MD, USA) was then used for cDNA synthesis according to the manufacturer’s instructions. The resulting cDNA sample was diluted 1:10 in H_2_O and used for PCR.

## Results

### The *pdu* Operon is Polycistronically Transcribed

To investigate the transcription of the *pdu* operon, promoters were predicted *in silico* using the BPROM (www.softberry.com) prediction tool, the nucleotide sequence of *S.* Typhimurium strain LT2 (NC_003197.1), and the 500-bp section upstream of the translational start site of each gene of the *pdu* operon. Possible promoters with probability scores of ≥20 were found in front of nearly all genes except *pduC, pduH, pduN, pduP, pduS*, and *pduX*. Binding sites of transcription factors were not found within these predicted promoter sequences, with the exception of P*_pduA_* to which several transcription factors putatively bind.

**FIGURE 1:**
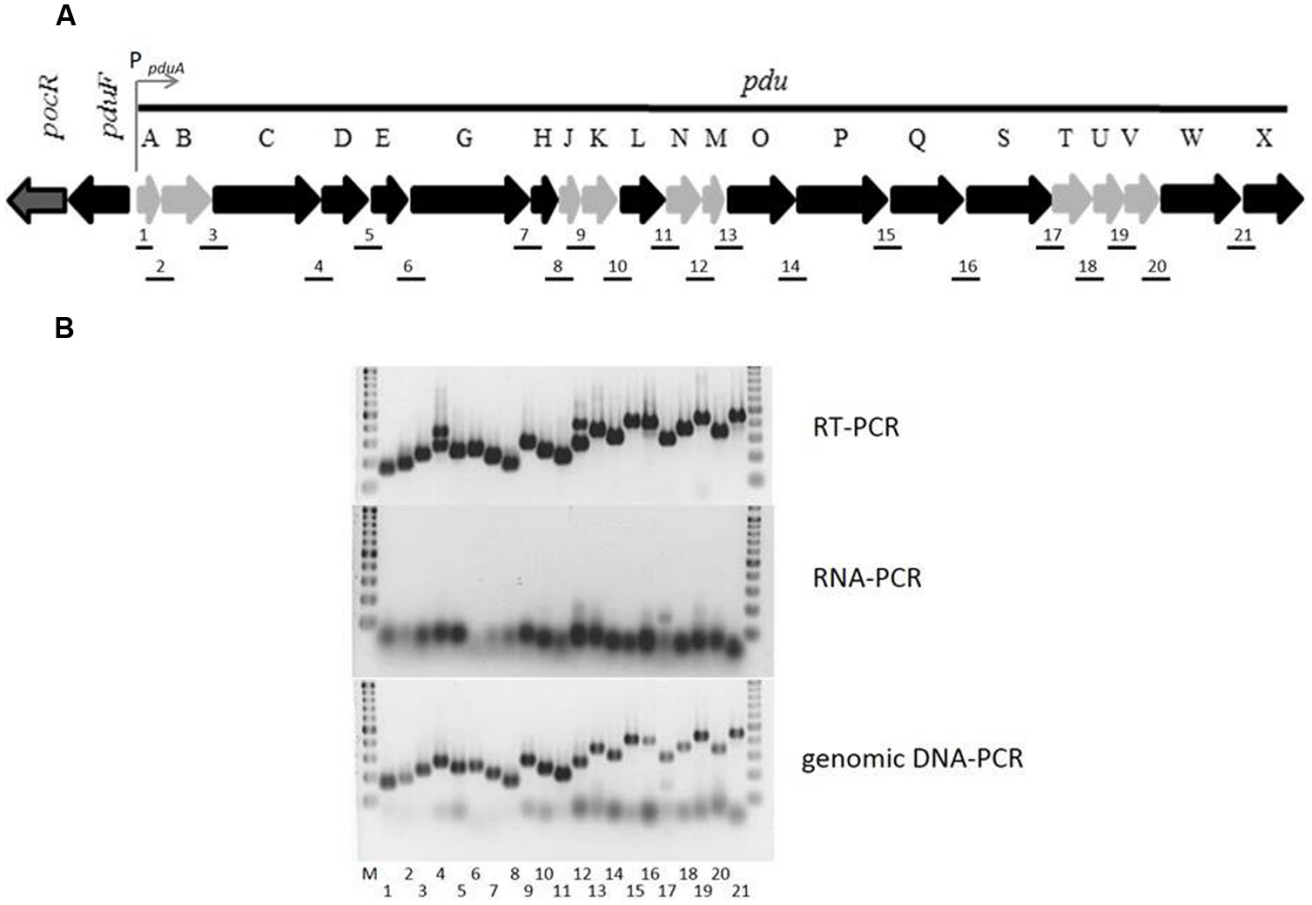
**Genomic organization and transcription of the *pdu* operon in *Salmonella enterica* serovar Typhimurium (*S.* Typhimurium) strain 14028. (A)** The *pdu* genes coding for proteins with enzymatic function are colored in black, and genes-encoding proteins contributing to the formation of the microcompartment-like polyhedral body are shaded in gray. The promoter P*_pduA_* is indicated with an arrow upstream of *pduA.* **(B)** Transcription of the *pdu* operon. For RNA isolation, strain 14028 was grown aerobically in VB-NCE-YE medium supplemented with trace elements, cobalamin, and 50 mM 1,2-PD to an OD_600_ of 2.0. An aliquot of 100 ng of cDNA obtained by reverse transcription of RNA was used as a template for RT-PCR. Genomic DNA (100 ng) of strain 14028 served as a positive control, and total RNA (100 ng) as a negative control. Line numbers match the PCR product numbers in **(A)**. GeneRuler^TM^ DNA ladder mix (Thermo Scientific) was used as a marker (M). The resulting fragments were separated on 2% (w/v) agarose gels.

To test whether the *pdu* operon (**Figure 1A**) is transcribed as a single polycistronic mRNA from P*_pduA_* or as multiple mRNAs from different promoters, RNA was isolated from *S.* Typhimurium grown with 1,2-PD and used as a template for reverse transcriptase polymerase chain reaction (RT-PCR) with random oligonucleotides. The RNA was shown to be free of DNA. Amplicons spanning the intergenic regions between the 3′-end of the upstream and the 5′-end of the downstream adjacent *pdu* genes were generated with all primer pairs using cDNA as a template, and the lengths of these amplicons corresponded to the PCR products obtained with genomic DNA as the template (**Figure 1B**). It can thus be concluded that the *pdu* operon is transcribed as a single mRNA, confirming the single transcription start site of the *pdu* operon (Chen et al., 1995).

### *Pdu* Gene Expression during Growth of *S.* Typhimurium with 1,2-PD

**FIGURE 2:**
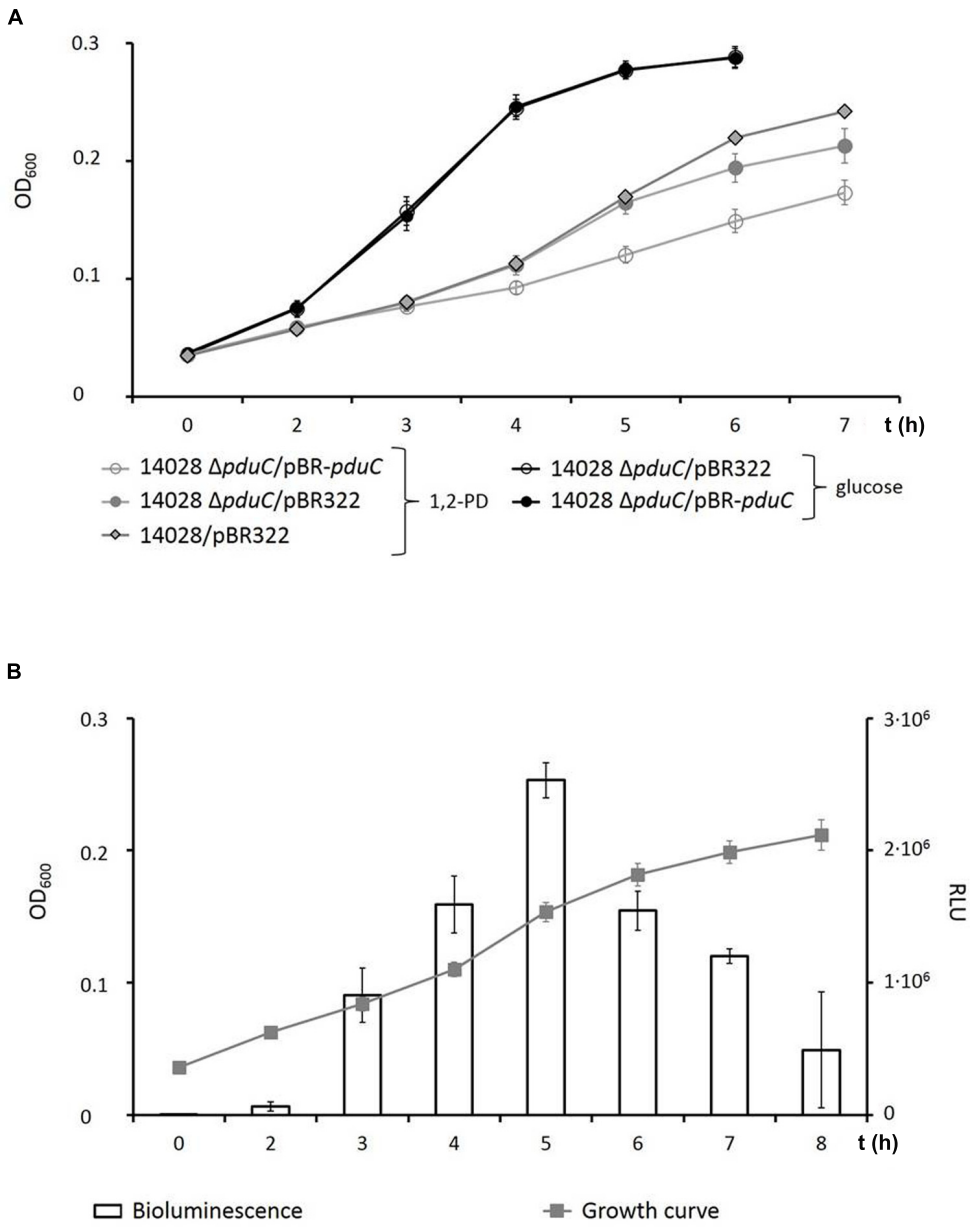
**Growth assays of strain 14028 with 1,2-PD. (A)** Strains 14028/pBR322, 14028 Δ*pduC*/pBR322, and 14028 Δ*pduC* /pBR-*pduC* were cultivated in 50-ml cultures of VB-NCE-YE medium supplemented with trace elements, 40 mM tetrathionate and 25 mM 1,2-PD (gray traces), or 27.8 mM glucose as control (black traces) in an oxygen-free environment. Samples were taken at the indicated time points and transferred to microtiter plates to monitor the OD_600_. **(B)** The transcriptional activity of *pduA* was determined using reporter strain 14028 P*_pduA_*::*lux*. OD_600_ and RLU_490_ were measured in parallel. Standard deviations of three independent experiments are indicated.

Under anaerobic conditions, *S.* Typhimurium strain 14028 can be grown in VB-NCE-YE medium supplemented with 1,2-PD and tetrathionate as an electron acceptor (Price-Carter et al., 2001). To investigate whether growth of *S.* Typhimurium indeed depends on the expression of the *pdu* operon under these conditions, the gene *pduC*, which encodes the large subunit of diol dehydratase PduC, the central enzyme in 1,2-PD degradation, was deleted. Growth of strain 14028 Δ*pduC* with 1,2-PD was severely attenuated under anaerobic conditions (**Figure 2A**), thus confirming the analysis of a *pduC* point mutation (Walter et al., 1997). The deletion was partially restored when the complementation plasmid pBR-*pduC* was present in 14028 Δ*pduC*. To demonstrate that this growth phenotype of 14028 Δ*pduC* is specific to a medium with 1,2-PD as carbon source, the strains were also grown anaerobically in the presence of glucose. No significant differences between the two growth curves were observed (**Figure 2A**). Growth of *S.* Typhimurium with 1,2-PD resulted in a lower cell density than growth with the better energy source glucose.

A stimulatory effect of 1,2-PD on the *pdu* genes has been demonstrated by Mu d-*lac* transcriptional fusions (Rondon and Escalante-Semerena, 1992). For more detailed investigation, the expression of the *pdu* operon was monitored during all growth phases using the reporter strain 14028 P*_pduA_*::*lux*, which was cultivated under the same growth conditions as described above. Bioluminescence (RLU_490_) was recorded in parallel with OD_600_ (**Figure 2B**). As a control, the same strain was grown with glucose, and a maximal value of 2,000 RLU_490_ was determined as background luminescence or leaky expression of the reporter. The transcriptional activity of P*_pduA_* reached a maximum of 2.53 × 10^6^ RLU_490_ in approximately the middle of the logarithmic growth phase, and then gradually decreased until the culture reached the stationary phase 8 h after inoculation.

### 1,2-PD at 50 μM is Sufficient to Induce P*_pduA_*, and Glucose Represses *pdu* Transcription

**FIGURE 3:**
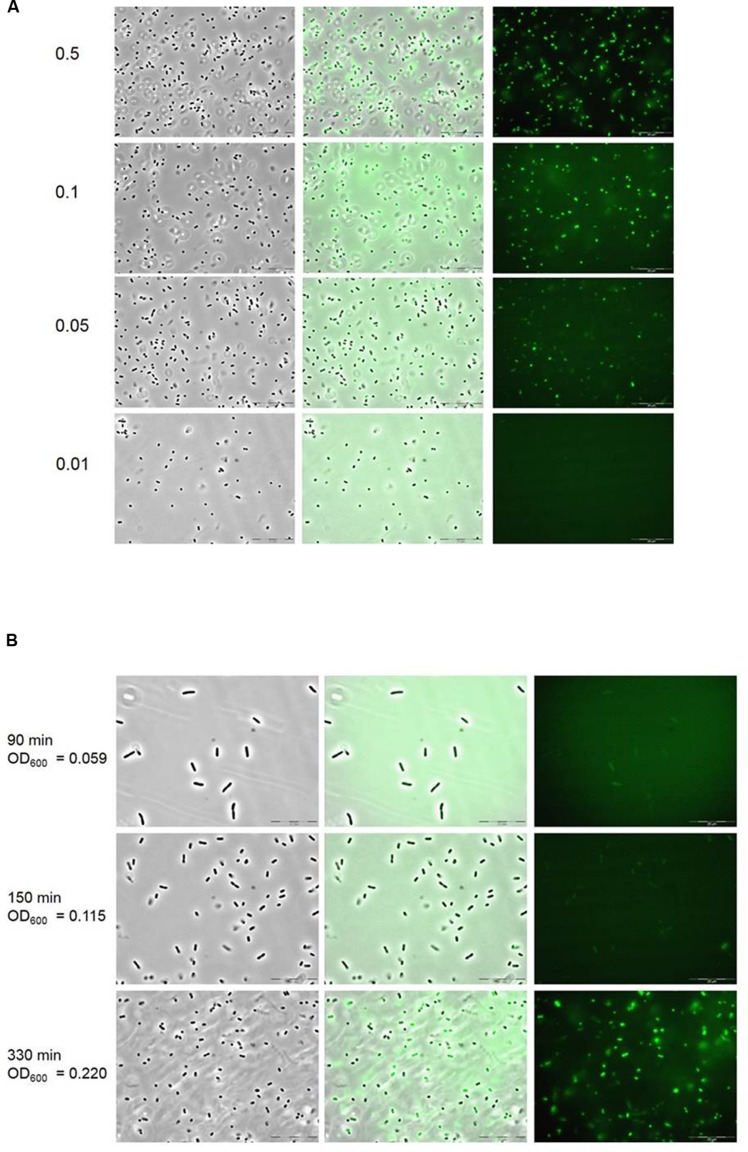
**Microscopic survey of strain 14028 P*_pduA_*::*gfp* at varying concentrations of 1,2-PD and glucose. (A)** Images were obtained 270 min post-inoculation in VB-NCE-YE medium supplemented with trace elements, tetrathionate, and 0.5, 0.1, 0.05, and 0.01 mM 1,2-PD as indicated on the left. The OD_600_ of the samples ranged from 0.143 to 0.151. **(B)** Repressing influence of glucose concentrations on the induction of P*_pduA_* by 1,2-PD. Cells were grown in the presence of 1 mM 1,2-PD and 1 mM glucose and monitored at the indicated cell densities and time points post-inoculation.

To determine the minimal concentration of 1,2-PD required to induce gene expression from P*_pduA_*, the reporter strain 14028 P*_pduA_*::*gfp* was grown anaerobically in VB-NCE-YE supplemented with trace elements, tetrathionate, and 1,2-PD in concentrations of 0.05 μM, 0.01 μM, 0.5 μM, 1 μM, 5 μM, 10 μM, 50 μM, 0.1 mM, 0.5 mM, and 1 mM. As a negative control, 14028 P*_pduA_*::*gfp* was grown in medium supplemented with glucose. Overnight cultures of 14028 P*_pduA_*::*gfp* were diluted 1:250 in the respective media and incubated at 37°C in the anaerobic chamber. Samples were taken every 30 min from time point 0 to 330 min and analyzed by fluorescence microscopy. Transcriptional activity of P*_pduA_* was observed at 1,2-PD concentrations of 50 μM and higher, but not at 10 μM or lower (**Figure 3A**).

To investigate the possible catabolite repression of P*_pduA_*, strain 14028 P*_pduA_*::*gfp* was anaerobically grown in VB-NCE-YE supplemented with tetrathionate, trace elements, and 1 mM 1,2-PD, and with increasing glucose concentrations of 1, 5, 10, and 25 mM. Cells were then monitored as described in the preceding experiment. At equimolar concentrations of 1,2-PD and glucose (1 mM), no fluorescence was observed until late exponential phase (OD_600_ = 0.220) due to glucose catabolization (**Figure 3B**). These results confirm the suggestion that the promoters involved in 1,2-PD utilization are repressed by glucose (Roth et al., 1996).

### FucA is Required for Proliferation of *S.* Typhimurium with Fucose

**FIGURE 4:**
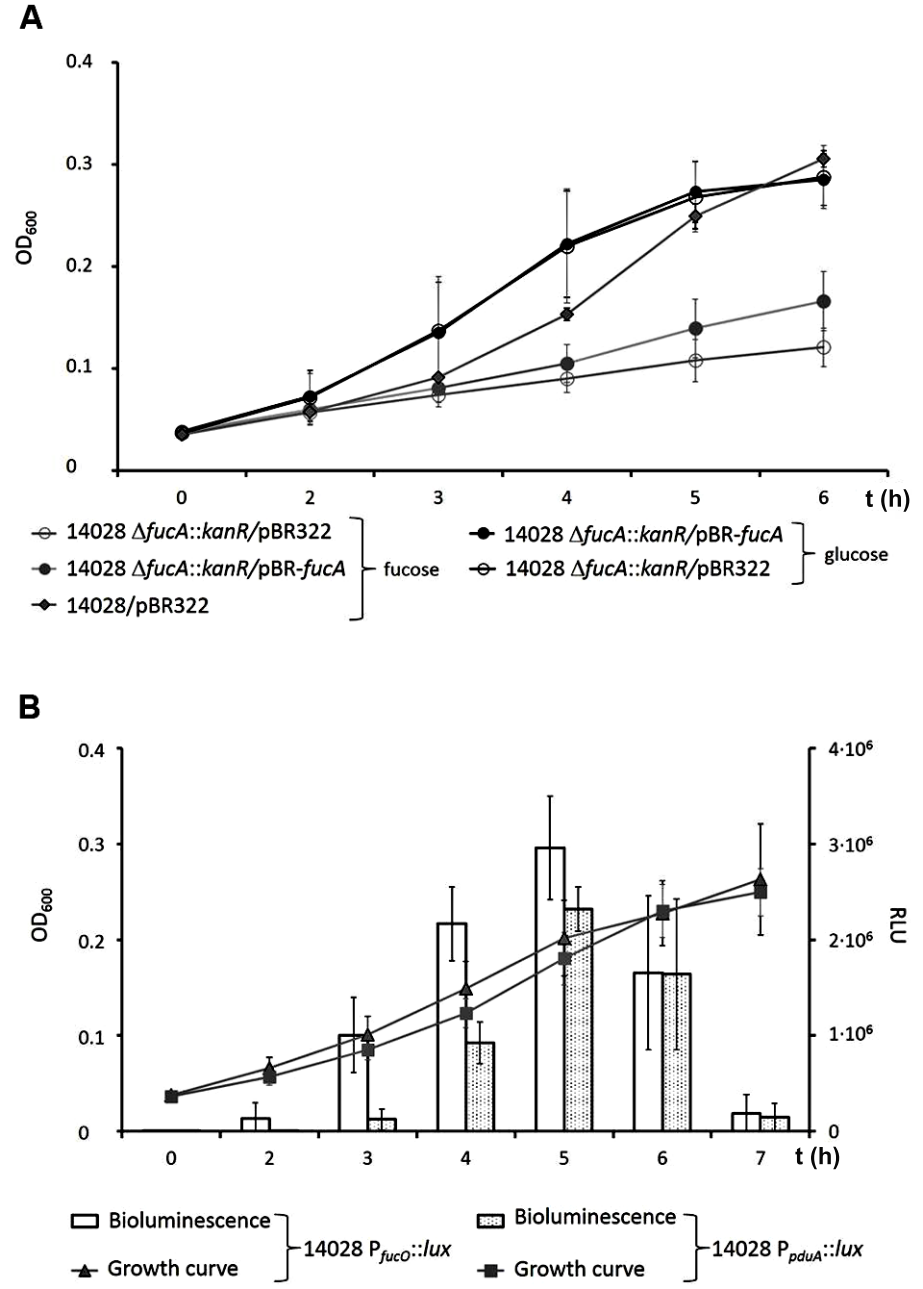
**Anaerobic growth of strain 14028 with fucose. (A)** Growth curves of 14028 Nal^R^, 14028 Δ*fucA*::*kan* and 14028 Δ*fucA*::*kan*/pBR-*fucA* with either 25 mM fucose (in gray) or 27.8 mM glucose (in black). Standard deviations of three independent experiments are indicated. **(B)** Transcriptional activity of P*_fucO_* and P*_pduA_* was visualized by monitoring bioluminescence of 14028 P*_fucO_*::*lux* and 14028 P*_pduA_*::*lux*.

Given that 1,2-PD is derived from the fermentative degradation of fucose, we investigated the anaerobic growth of *S.* Typhimurium strain 14028 in VB-NCE-YE medium containing fucose. Tetrathionate was also added to permit anaerobic respiration in the case of 1,2-PD formation and degradation. Growth of strain 14028 Δ*fucA*::*kanR*, in which *fucA* was replaced by a kanamycin resistance cassette, was severely attenuated when compared to that of strain 14028 (**Figure 4A**). To complement the replacement of *fucA*, mutant 14028 Δ*fucA*::*kanR* was equipped with pBR322-*fucA*. Although the plasmid did not completely restore the ability to use fucose, growth of the complemented mutant was improved from that of 14028 Δ*fucA*::*kanR*. As a control, the mutant and the complemented strain were grown in VB-NCE-YE with glucose to exclude pleiotropic effects due to the mutation procedure. Possible biphasic growth due to sequential 1,2-PD utilization as suggested earlier (Obradors et al., 1988) was not observed.

To compare the expression pattern of the *pdu* and *fuc* operon, strains with luciferase fusions to the promoters of *pduA* and *fucO* (P*_pduA_* and P*_fucO_*) were grown in VB-NCE-YE medium supplemented with 25 mM fucose, tetrathionate, and trace elements (**Figure 4B**). Luminescence of P*_fucO_*::*lux* was detected after 2 h and that of P*_pduA_*::*lux* at 3 h post-inoculation, suggesting a sequential induction of both operons according to their metabolic function. The reporter activity of both strains then increased until the late exponential phase of the culture and decreased after the culture reached the stationary phase. Notably, the transcriptional activity of P*_fucO_* was significantly higher than that of P*_pduA_* (*p* < 0.05). Both operons were transcribed simultaneously during the exponential growth phase, but the transcription of the *pdu* operon was slightly retarded when compared to that of the *fuc* genes. The data also showed that the 1,2-PD degrading factors were produced very early, even while those degrading fucose were still expressed, indicating that both substrates were simultaneously utilized.

### The *fuc* and *pdu* Operons are Highly Conserved among *S. enterica* Strains

A comprehensive database search was performed with BLAST using all proteins encoded by the *fuc* and *pdu* operon of *S.* Typhimurium strain LT2 (NC_003197) as queries. Proteins were considered to be absent from the respective serovar in the event of a query coverage below 60% and an identity score below 90%. Homologs of proteins encoded by the *pdu* and the *fuc* operon of strain LT2 were identified in all 83 investigated serovars of *S. enterica* ssp. *enterica* investigated here, with the exceptions of serovar Litchfield and Stanleyville for which, however, only a shotgun sequence is available (Supplementary Table S2). Homologs of both pathways were also found in the *S. enterica* subspecies *salamae*, *indica*, *diarizonae, arizonae*, and *houtenae.* These results indicate that the *pdu* and *fuc* operons are highly conserved and present in nearly all *Salmonella* strains sequenced thus far.

## Discussion

When enteropathogens enter the gastrointestinal tract, they encounter nutrient competition with the host and the commensal microbiota. This nutrient limitation may be overcome by specific metabolic adaptations that contribute to proliferation within distinct host compartments. The mucus layer is a potential source of nutrients for pathogens that colonize and eventually penetrate the gut epithelium. The mucus is composed of mucin oligosaccharides, which are characterized by a large variety of carbohydrate side-chains that also contain rhamnose and fucose.

As revealed in the present study by database querying, the *pdu* and *fuc* operons are highly conserved and ubiquitously present in *S. enterica* strains, indicating that their gene products play a role in the metabolic adaptation of salmonellae to their environments. This inference is supported by the observation that compounds of the 1,2-PD utilization pathway are produced in high amounts when *S.* Typhimurium is grown under *in vivo*-mimicking conditions and in murine macrophages (Adkins et al., 2006; Shi et al., 2006; Sonck et al., 2009). 1,2-PD is obtained by the degradation of rhamnose and fucose, two sugars that are common constituents of glycoconjugates not only of the gut mucus, but also of plant cell walls. In line with this observation, a transcriptome of *S*. Typhimurium strain SL1344 showed a marked upregulation of its *fuc*, *pdu*, *cob*/*cbi*, and ethanolamine utilization (*eut*) genes when the pathogen colonized lettuce and cilantro by co-infection with a plant pathogen (Goudeau et al., 2013). Interestingly, such a transcriptional activation of *pdu* genes has also been observed for *Listeria monocytogenes* in the intestines of mice (Toledo-Arana et al., 2009). The gut pathogen *Campylobacter jejuni* has been shown to use L-fucose, which is probably derived from mucin, during colonization in a piglet model (Stahl et al., 2011).

Taken together, we hypothesize that the ability to degrade fucose and anaerobically respire 1,2-PD, probably using tetrathionate as a terminal electron acceptor, provides an advantage to *S.* Typhimurium during competition with commensal bacteria to colonize the intestinal mucus layer.

## Conflict of Interest Statement

The authors declare that the research was conducted in the absence of any commercial or financial relationships that could be construed as a potential conflict of interest.
