# Supplementary material for: Regulation of fucose and 1,2-propanediol utilization by Salmonella enterica serovar Typhimurium
Source: Front Microbiol. 2015 Oct 12;6:1116. doi: 10.3389/fmicb.2015.01116 (PMC4600919; doi:10.3389/fmicb.2015.01116)
Supplement: Supplementary file 1 [file Table_1.DOCX]

**Table S1.** Oligonucleotides used in this study.

| **Primer** | **Sequence (5'-3')** | **Target** |  |  |
| --- | --- | --- | --- | --- |
| **Primers for RT-PCR** |  |  |  |  |
| 24_*pduA*_RT_F | CAGCCAATGTGATGTTAGTG | *pduA* | |  |
| 25_*pduA*_RT_R | TTCTACATCGGTGTGAGG | *pduA* | |  |
| 26_*pduAB*_RT_F | CCTCACACCGATGTAGAA | *pduAB* | |  |
| 27_*pduAB*_RT_R | GACAAATTCCGTTAAACTGC | *pduAB* | |  |
| 28_*pduBC*_RT_F | CACCAGCTTTAGTAACGAAGC | *pduBC* | |  |
| 29_*pduBC*_RT_R | CAAAGCCGTCCTGATTCAC | *pduBC* | |  |
| 32_*pduCD*_RT_F | CGCTGGGAAGAGATTAAAAAC | *pduCD* | |  |
| 33_*pduCD*_RT_R | CGACGGCGATAATGACTTC | *pduCD* | |  |
| 34_*pduDE*_RT_F | GAAGTACCAGGCAAAGTCG | *pduDE* | |  |
| 35_*pduDE*_RT_R | GTAGTCGCTGACCTTTGC | *pduDE* | |  |
| 48_*pduEG*_RT_F | GTATCGTTCAACGAAAGAAG | *pduEG* | |  |
| 49_*pduEG*_RT_R | CTGTGTGTAATCGTCAGTG | *pduEG* | |  |
| 50_*pduGH*_RT_F | CGGGCGAGGAAATATTCG | *pduGH* | |  |
| 51_*pduGH*_RT_R | GATGCTGAAGCAGGAAAGG | *pduGH* | |  |
| 52_*pduHJ*_RT_F | CGCAACACCGGTAATAACG | *pduHJ* | |  |
| 53_*pduHJ*_RT_R | GTACGTTGGCGGATTTAACC | *pduHJ* | |  |
| 54_*pduJK*_RT_F | GATGTTGAGGCCATTTTACC | *pduJK* | |  |
| 55_*pduJK*_RT_R | CAGGCCATTCCACTGTTC | *pduJK* | |  |
| 56_*pduKL*_RT_F | GAATCAGAACTGGTCAGCTG | *pduKL* | |  |
| 57_*pduKL*_RT_R | CAGCCGCTCATAATCCTG | *pduKL* | |  |
| 58_*pduLM*_RT_F | CACATTGATACCGATGAAGC | *pduLM* | |  |
| 59_*pduLM*_RT_R | GCTGACAGAACAGTGCTG | *pduLM* | |  |
| 60_*pduMN*_RT_F | GCAAATGCATCGTGACCG | *pduMN* | |  |
| 61_*pduMN*_RT_R | CCGCTGAGCAAAACCAGTTC | *pduMN* | |  |
| 62_*pduNO*_RT_F | CAAATGAGGCCATTGACCTC | *pduNO* | |  |
| 63_*pduNO*_RT_R | CTTCCAGGGCCGAAATCTC | *pduNO* | |  |
| 64_*pduOP*_RT_F | GCGACGGCATATTAATTGGG | *pduOP* | |  |
| 65_*pduOP*_RT_R | CATCGCGCTGATAATGGC | *pduOP* | |  |
| 66_*pduPQ*_RT_F | CACGCTGCAAACCTCGATATTC | *pduPQ* | |  |
| 67_*pduPQ*_RT_R | CTGAACCACTGTGTGGATG | *pduPQ* | |  |
| 68_*pduQS*_RT_F | CAATGATATCGCGGCGATC | *pduQS* | |  |
| 69_*pduQS*_RT_R | CTTTGAGCATCGGTTCACATTC | *pduQS* | |  |
| 70_*pduST*_RT_F | CAGCATATTGGTGCCAGC | *pduST* | |  |
| 71_*pduST*_RT_R | TTCCCCGGACAGATGGTC | *pduST* | |  |
| 72_*pduTU*_RT_F | CAATAACGCCGTGACGGTTG | *pduTU* | |  |
| 73_*pduTU*_RT_R | GGCACCGGTAAAGCGATC | *pduTU* | |  |
| 74_*pduUV*_RT_F | GTTTCTGCCGTCGAGTATGC | *pduUV* | |  |
| 75_*pduUV*_RT_R | CCGCAACCAGCGAAATACG | *pduUV* | |  |
| 76_*pduVW*_RT_F | GCGCACAGCAGATTTTTATTACC | *pduVW* | |  |
| 77_*pduVW*_RT_R | CAATGTCCCGCAGACTGTTAATG | *pduVW* | |  |
| 78_*pduWX*_RT_F | CATTTATCCAGACGGAGAACG | *pduWX* | |  |
| 79_*pduWX*_RT_R | CACATCGACGCGAATCTCG | *pduWX* | |  |
| **Deletion primers** |  |  | |  |
| 40_*pduC*_del_F | GAAGCACTGGCGAAACGCCCTGTGA  ATCAGGACGGCTTTGTTAAGGAGTG  GTGTAGGCTGGAGCTGCTTC | *pduC* (32 bp upstream of the ATG start codon plus first 18 bp of the coding region) | | |
| 41_*pduC*_del_R | GTTTTTAATCTCTTCCCAGCGTTCTCC  CTGCAGGCGATAACCTGTTGCCG  CATATGAATATCCTCCTTA | *pduC* (last 36 bp of the coding sequence plus 14 bp downstream of the stop codon) | | |
| 90_*fucA*_del_F | GCGCGGCAGATTATTGATACCTGTCT  GGAAATGACGCGGTTAGGGTTAAA  GTGTAGGCTGGAGCTGCTTC | *fucA* (see above) | | |
| 91_*fucA*_del_R | CTCCAGTACGATAGCGATCGCCTCGT  CATCCAGTACCGGTACCGGATCGA  CATATGAATATCCTCCTTA | *fucA*(see above) | | |
| 92_*fucA*_conf_F | AATTATTGCCACGAAAACGG | *fucA* | | |
| 93_*fucA*_conf_R | TGTCACTTTATCGACCACAC | *fucA* | | |
| 2043_*kan*_R ^3^ | CGATGCCTGCTTGCCG | *kan* | | |
| **Cloning of pUTs-promoter fusions** | |  | | |
| 14_P*_fucO_*_*Sac*I_F | GAGGAGCTCTGTCTTTATTGATG  CTGACGG | P*_fucO_* | | |
| 15_P*_fucO_*_*Sma*I_R | GAGCCCGGGCGCCTGTCTCCTG  AC | P*_fucO_* | | |
| 22_P*_pduA_*_*Sac*I_F | GAGGAGCTCCACCTTCTGAGCA  AAGTTCG | P*_pduA_* | | |
| 23_P*_pduA_*_*Kpn*I_R | GAGGGTACCGTTGGGACTATAA  GAAGATGC | P*_pduA_* | | |
| 103_P*_pduA_*_*Not*I_R | ATAAGAATGCGGCCGCGTTGG  GACTATAAGAAGATGC | P*_pduA_* | | |
| 105_P*_pduA_*_*Not*I_F | ATAAGAATGCGGCCGCCACCT  TCTGAGCAAAGTTCG | P*_pduA_* | | |
| 3545_*luxC*59_R | GATTGCACTAAATCATCACTTTC  GG | *luxC* (pUTs) | | |
| 3123_*luxC*1_R | CATAAGGCAATATTTGCTC | *luxC* (pUTs) | | |
| 3696_pUTs-Test_F | TGGAATTCTGACTCTTATAC | pUTs | | |
| 3697_pUTs-Test_R | CAGGTATTTATTCGGCGC | pUTs | | |
| **Cloning of pBR322 complementation plasmids** | |  | | |
| 96_*fucA*_compl_F_  *Pst*I | AACTGCAGATCCTCATCGGTTCA  GAAG | P *_fucA_*+ *fucA* | | |
| 97_*fucA*_compl_R_  *Ahd*I | GAGGACTCCCCGTCAATCATTCT  GTTCGCCATC | *fucA* | | |
| 98_pBR_Amp_F | ACCAGTCACAGAAAAGCATC | pBR-Amp^R^ | | |
| 99_pBR_Amp_R | CAGTTACCTTCGGAAAAAGA | pBR-Amp^R^ | | |
| 83_Lig_*pduC*::pBR_R | GTTAAGGGATTTTGGTCATG | pBR | | |
